# Supplementary material for: An FGFR3/MYC positive feedback loop provides new opportunities for targeted therapies in bladder cancers
Source: EMBO Mol Med. 2018 Feb 20;10(4):e8163. doi: 10.15252/emmm.201708163 (PMC5887543; doi:10.15252/emmm.201708163)
Supplement: Supplementary file 1 — Appendix [file EMMM-10-e8163-s001.pdf]

Appendix: Supplementary figures and legends

An FGFR3/MYC positive feedback loop provides new opportunities for targeted therapies in bladder cancers

Mélanie Mahe[1,2,#], Florent Dufour[1,2,#], Hélène Neyret-Kahn[1,2], Aura Moreno-Vega[1,2], Claire Beraud[3], Mingjun Shi[1,2], Imene Hamaidi[4], Virginia Sanchez-Quiles[1,2], Clementine Krucker[1,2], Marion Dorland-Galliot[1,2], Elodie Chapeaublanc[1,2], Remy Nicolle[1,2], Hervé Lang [4], Celio Pouponnot[5,6, 7], Thierry Massfelder[8], François Radvanyi[1,2,] and Isabelle Bernard-Pierrot[1,2,*]

[1]Institut Curie, PSL Research University, CNRS, UMR144, Equipe Labellisée Ligue contre le Cancer, 75005 Paris, France
[2]Sorbonne Universités, UPMC Université Paris 06, CNRS, UMR144, 75005 Paris, France
[3] UROLEAD SAS, School of Medicine, 67085 Strasbourg, France
[4]Hôpitaux Universitaires de Strasbourg, Nouvel Hôpital Civil, Department of Urology, 67091 Strasbourg, France
[5] Institut Curie, Centre Universitaire, Bâtiment 110, 91405 Orsay cedex, France
[6] CNRS UMR3347
[7] INSERM U1021
[8]INSERM UMR_S1113, Section of Cell Signalization and Communication in Kidney and Prostate Cancer, INSERM and University of Strasbourg, School of Medicine, Fédération de Médecine Translationnelle de Strasbourg (FMTS), 67085 Strasbourg, France
#, these two authors contributed equally to this work

Table of Contents

**Appendix figure S1**	*FGFR3* and *MYC* expression in human bladder tumors.
**Appendix figure S2**	The PDX model F659 bearing a FGFR3-S249C is sensitive to FGFR3 inhibition with a pan-FGFR inhibitor.
**Appendix figure S3**	*MYC* expression decreased considerably following treatment with a pan-FGFR inhibitor. Western blot comparing FGFR3 and MYC levels in RT112 cells transfected for 72 h with FGFR3 siRNA, MYC siRNA or both.
**Appendix figure S4**	Enrichment of the FGFR3 locus in MYC and histone activation marks.
**Appendix figure S5**	MYC Enrichment on the FGFR3 locus in multiple tissues and in MYC-activated cell lines.
**Appendix figure S6**	The growth of aberrantly activated FGFR3-expressing cell xenografts and PDX is inhibited by pan-FGFR inhibitors.
**Appendix figure S7**	The p38 MAP kinase and PI3 kinase pathways are critical for NIH-3T3 cell transformation by FGFR3-S249C.
**Appendix figure S8**	Enrichment of the MYC locus in histone activation marks.
**Appendix figure S9**	Effect of combinations of FGFR inhibitors and BET inhibitors on RT112 and MGH-U3 cell viability.

Appendix figure S1

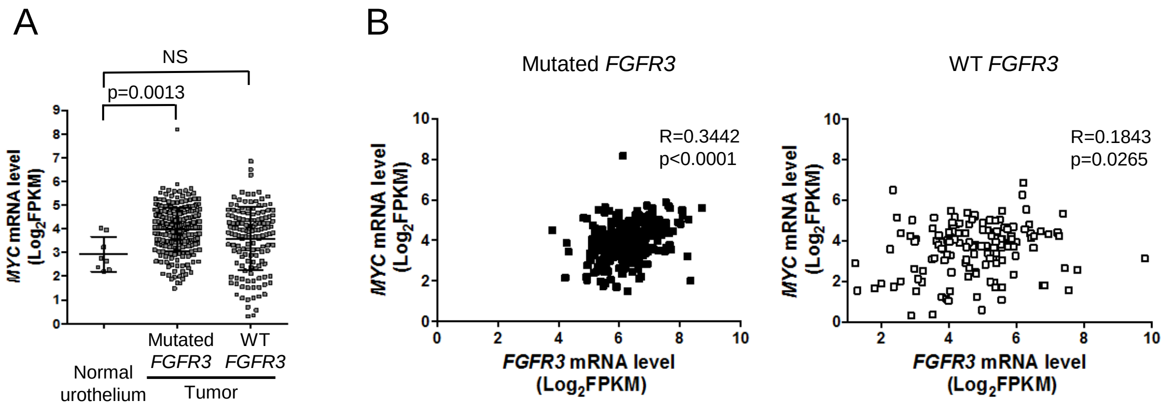

Appendix Figure S1: *FGFR3* and *MYC* expression in human bladder tumors. **(A)** *MYC* mRNA levels in normal human urothelium (*n*=8) and in human bladder tumors bearing *FGFR3* mutations (*n*=271) or wild-type *FGFR3* (*n*=145). Means and standard errors are represented. The significance of differences was assessed in Mann-Whitney tests.

(B) *MYC* and *FGFR3* mRNA levels in human bladder tumors harboring either mutated *FGFR3* (upper panel) or wild-type *FGFR3* (lower panel). Spearman's coefficient and *p*-values are indicated for the correlations between *MYC* and *FGFR3* mRNA levels in each group.

Appendix figure S2

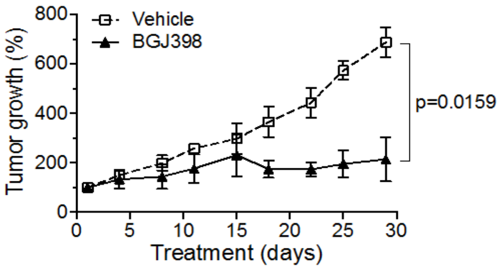

Appendix figure S2: the PDX model F659 bearing a FGFR3-S249C is sensitive to FGFR3 inhibition with a pan-FGFR inhibitor.
Effect of BGJ398 (oral gavage, 30 mg/kg/day), a pan-FGFR inhibitor, on the growth of PDX tumors bearing the FGFR3-S249C mutation. (*n*=5 animals/group). Data are presented as means ± SEM. Results were compared in Mann-Whitney test.

Appendix figure S3

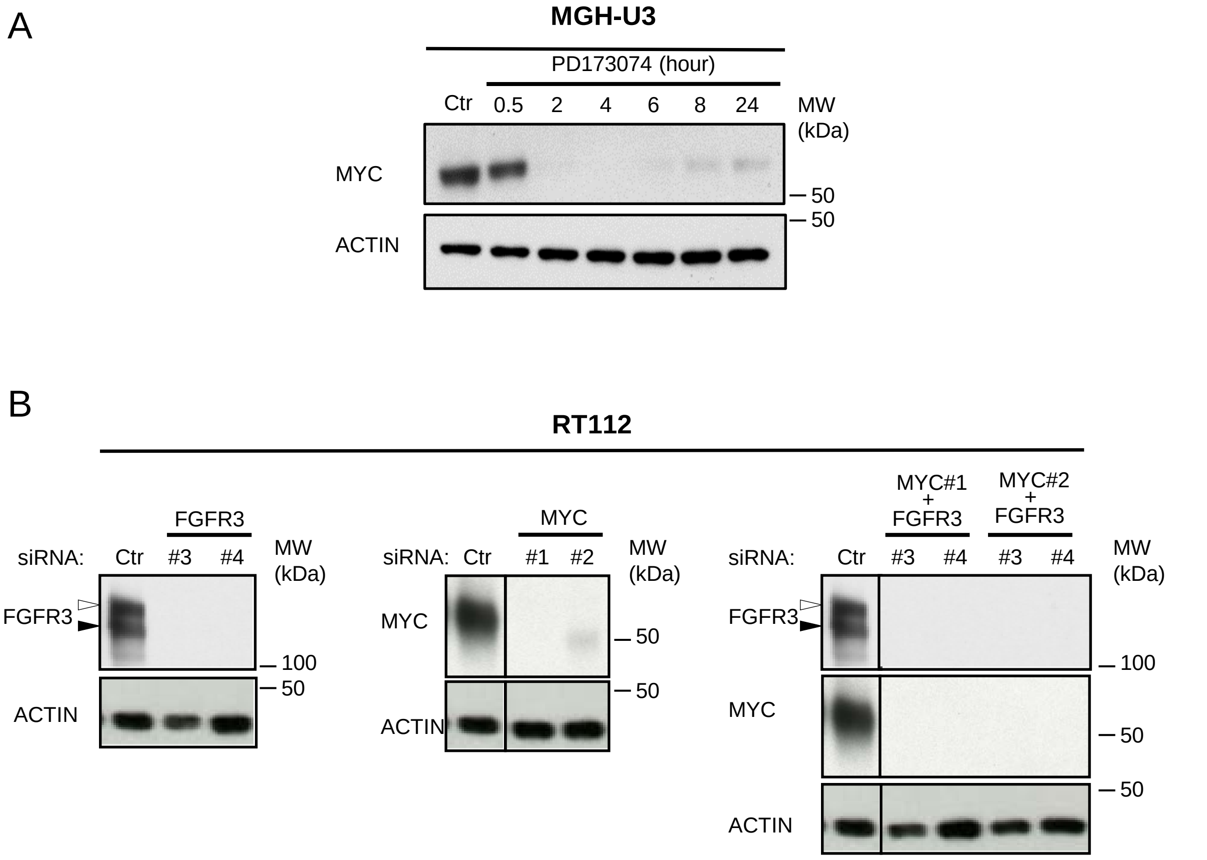

Appendix figure S3:

(A) *MYC* expression decreased considerably following treatment with a pan-FGFR inhibitor. MGH-U3 cells were treated with DMSO or 500 nM PD173074. Lysates were obtained at the indicated time points and MYC levels were analyzed by western blotting. Anti-actin antibody was used as a loading control.

(B) Western blot comparing FGFR3 and MYC levels in RT112 cells transfected for 72 h with *FGFR3* siRNA, *MYC* siRNA or both.

Appendix figure S4

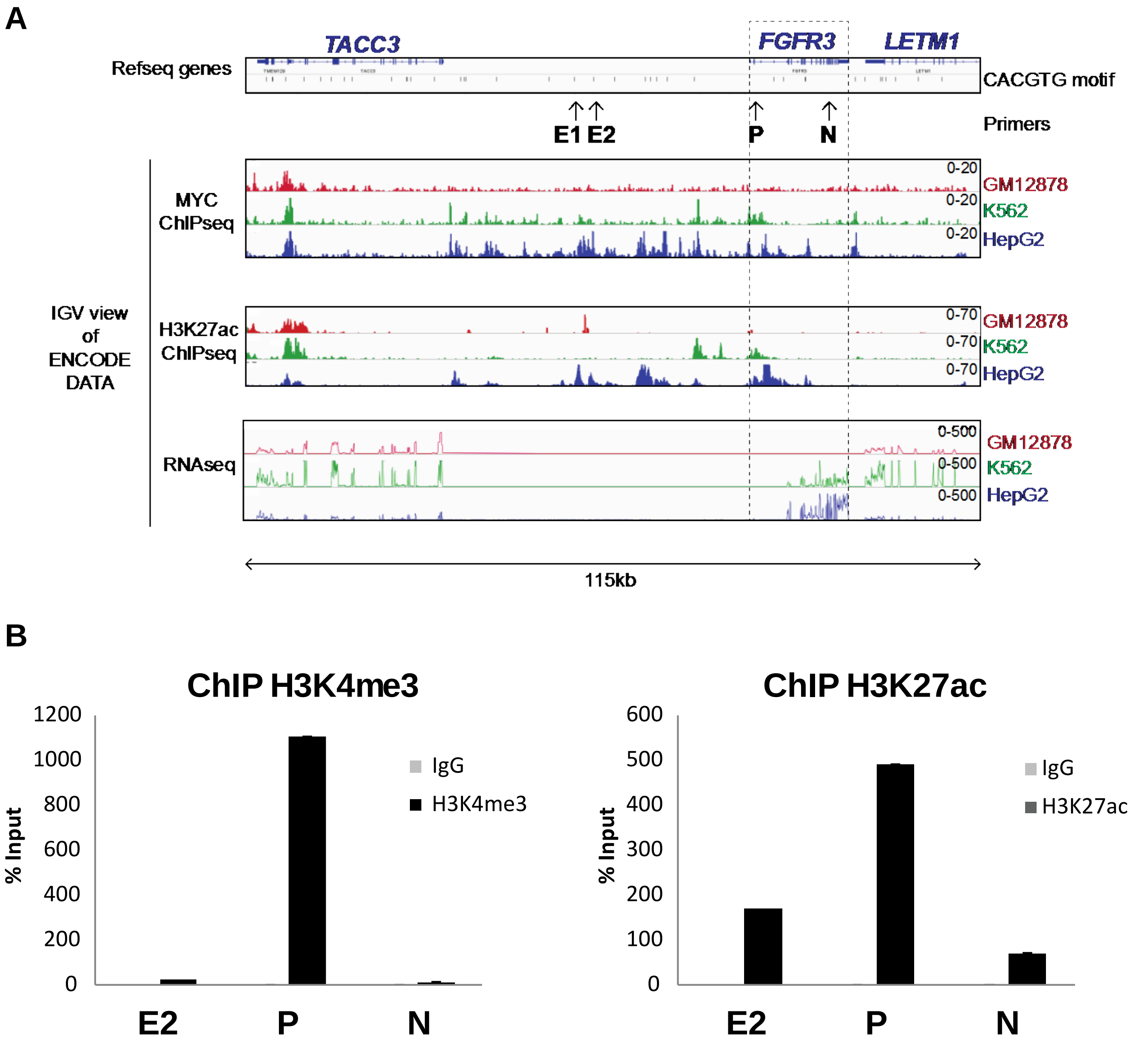

Appendix figure S4: Enrichment of the *FGFR3* locus in MYC and histone activation marks

(A) IGV view of selected ENCODE data for the region around the *FGFR3* locus. The following are indicated: primers binding to the region (E1, E2 in potential enhancers, P in the promoter and N in the negative region), binding motifs for MYC (CACGTG), ENCODE ChIPseq data for MYC, H3K27ac, and ENCODE RNAseq data for three cell lines.

(B) ChIP qPCR in the RT112 cell line for H3K27ac (mark of activated promoter and enhancer) and H3K4me3 (mark of activated promoters).

Appendix figure S5

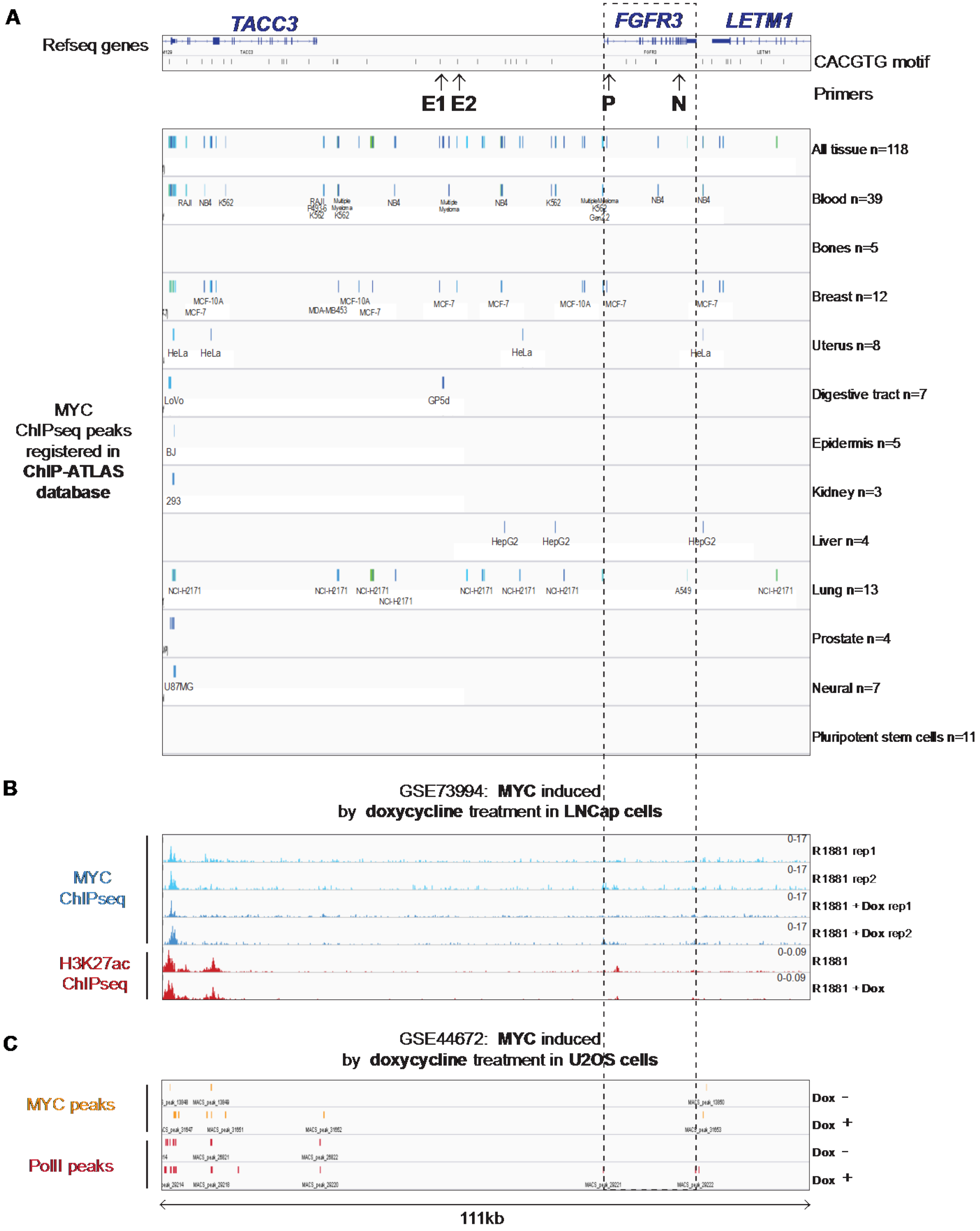

Appendix figure S5: MYC Enrichment on the *FGFR3* locus in multiple tissues and in MYC-activated cell lines

(A) IGV view of 118 MYC ChIP-seq peaks downloaded from the ChIP-Atlas database http://chip-atlas.org/) for the *FGFR3* locus (significance threshold = 50). The locations of the primers used for ChIP qPCR are indicated (E1, E2, P and N), together with MYC DNA-binding motifs (CACGTG). MYC peaks are shown merged (all tissues) or according to tissue type. The number of ChIPseq results included for each track is indicated (n).

(B) IGV view of a selected ChIPseq dataset from GEO series GSE73994. ChIPseq for MYC and PolII was performed in LNCaP cells stably transfected with inducible MYC. Cells were treated with the synthetic androgen R1881 or with R1881+doxycycline (to induce c-Myc overexpression)(Barfeld et al, 2017).

(C) IGV view of MYC and PolII ChIPseq peaks from GEO series GSE44672. MYC expression was induced with doxycycline in U2OS cells stably transfected with a doxycycline-inducible vector system encoding a human MYC complementary DNA (Walz et al, 2014).

Appendix figure S6

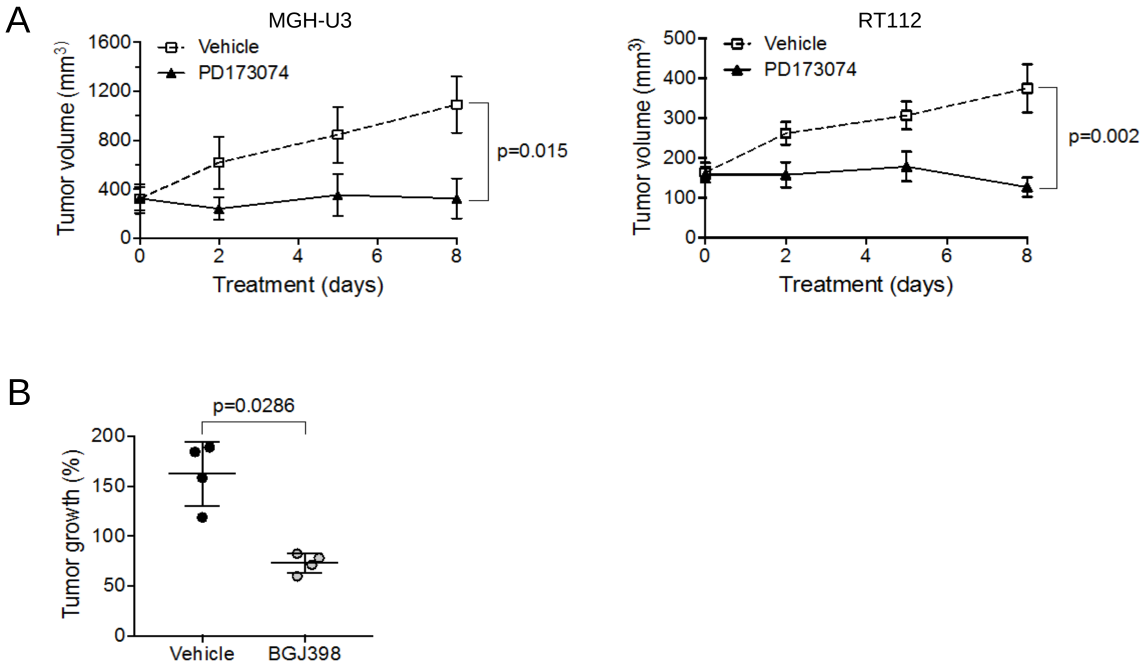

Appendix figure S6: The growth of aberrantly activated FGFR3-expressing cell xenografts and PDX is inhibited by pan-FGFR inhibitors.

(A) Effect of PD173074, a pan-FGFR inhibitor (oral gavage, 25 mg/kg, once daily, 6 days per week), on the growth of xenografts of MGH-U3 and RT112 bladder cancer cells expressing constitutively activated FGFR3-Y375C and FGFR3-TACC3 receptors, respectively (*n*=4 animals/group, two xenografts per animal (one in each flank)). Data are presented as means ± SEM. Results were compared in Mann-Whitney test.

(B) Effect of BGJ398 (oral gavage, 30 mg/kg/day for 4 days), a pan-FGFR inhibitor, on the growth of PDX bearing FGFR3-S249C mutation. (*n*=4 animals/group). Data are presented as means ± SEM. Results were compared in Mann-Whitney test.

Appendix figure S7

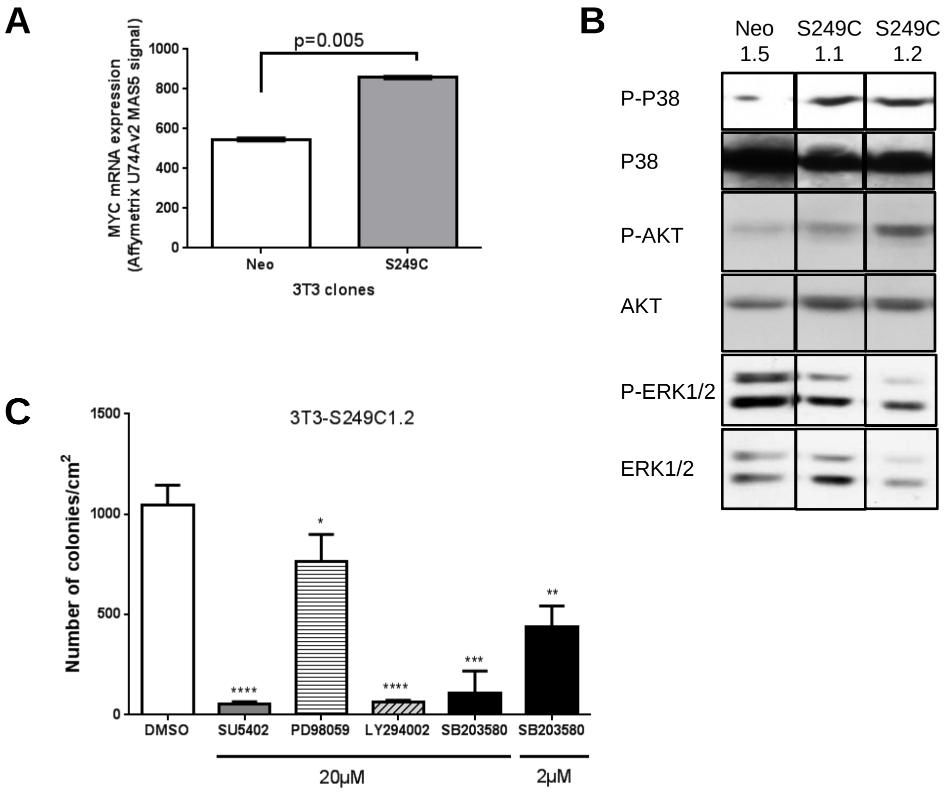

Appendix figure S7: The p38 MAP kinase and PI3 kinase pathways are critical for NIH-3T3 cell transformation by FGFR3-S249C

(A) *MYC* mRNA levels were measured in triplicate in control cells (Neo1.5 and Neo2.3) and in FGFR3-S249C-expressing cells (S249C1.1 and S249C 1.2), with an Affymetrix U74Av2 DNA array. Microarrays were scanned and intensities were calculated for each probe set, with the Affymetrix MAS 5.0 default settings. Results were compared in unpaired Student's *t*-tests.

(**B**) NIH-3T3 control cells (Neo1.5) and FGFR3-S249C-expressing cells (S249C1.1, S249C1.2) were starved of serum for 24 h before lysis. Western-blot analysis of 50 µg of whole-cell lysate protein with the following specific antibodies: anti-phospho-p38 (P-p38), anti-p38, anti-phospho-Akt (P-Akt), anti-Akt, anti-phospho-ERK1/2 and anti-ERK1/2 antibodies.

(C) Soft agar assay of FGFR3-S249C-expressing cells (S249C1.2) in the presence or absence of inhibitors. SU5402, PD98059, LY294002 and SB203580 are specific for FGFRs, MEK, PI3 kinase and p38 activation, respectively. The data shown are the means of two independent experiments carried out in triplicate and the standard errors are indicated. Results were compared in unpaired Student's *t*-tests, *, $p<0.05$; **, $0.001<p<0.005$; ***, $0.0001<p<0.001$; ****, $p<0.0001$.

Appendix figure S8

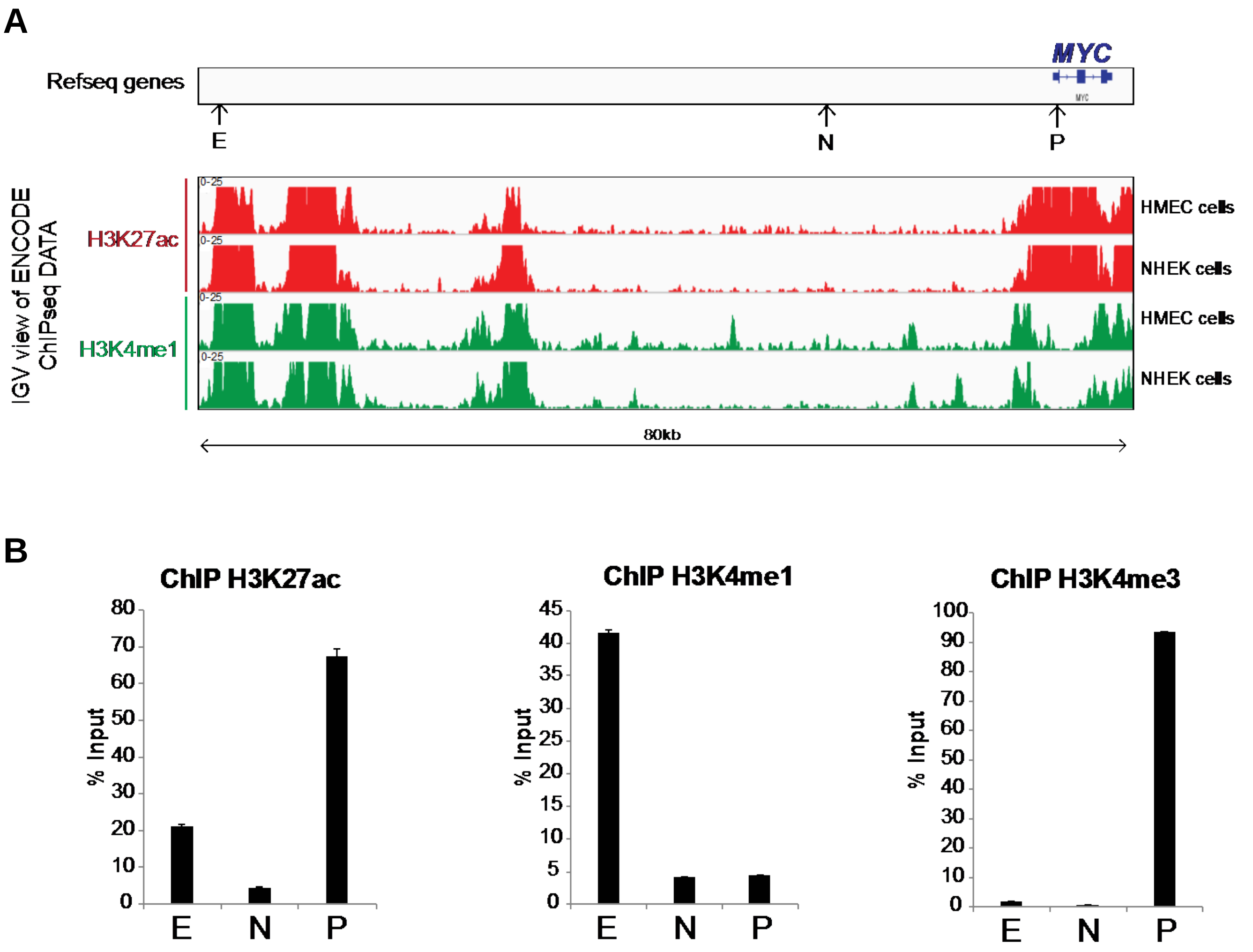

Appendix figure S8: Enrichment of the *MYC* locus in histone activation marks
(A) IGV view of selected ENCODE data for the region around the *MYC* locus. The following are indicated: primers binding to the region (E in a potential enhancer, P in the promoter and N in a negative region), and ENCODE ChIPseq data for H3K4me1 and H3K27ac in two epithelial cell lines (HMEC and NHEK).
(B) ChIP-qPCR data for the H3K27ac, H3K4me1 and H3K4me3 marks in the RT112 cell line.

Appendix figure S9

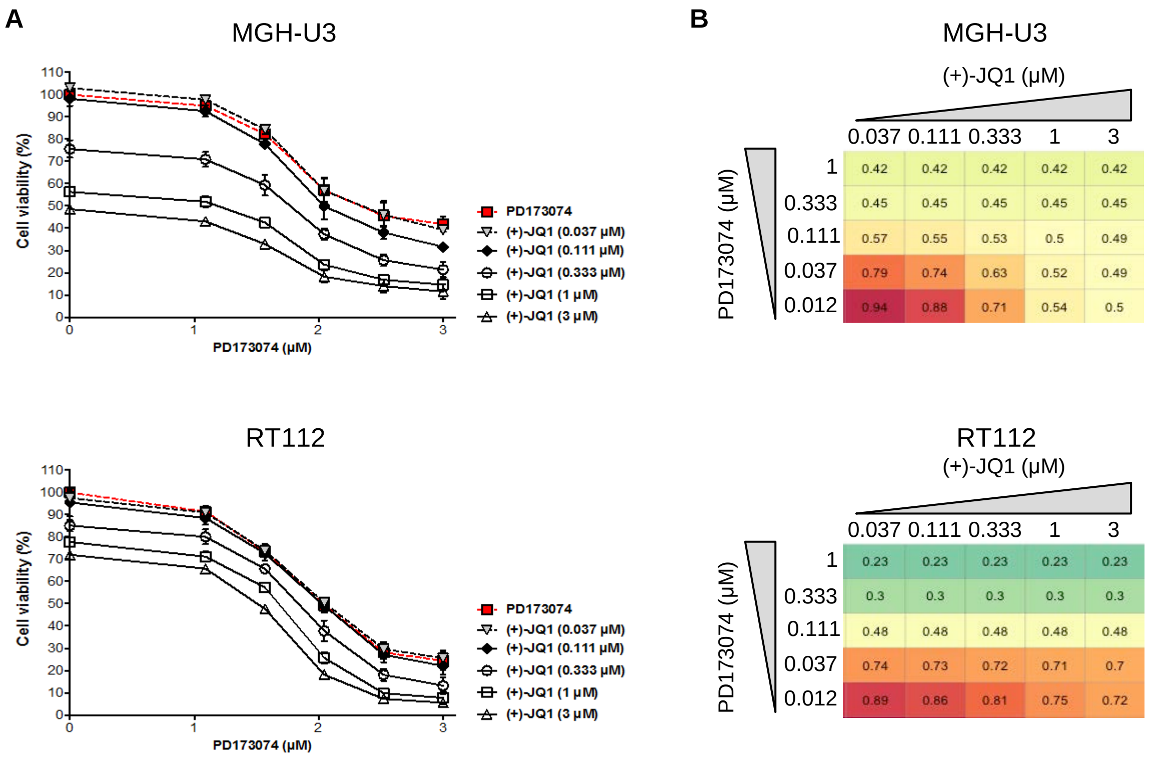

Appendix figure S9: Effect of combinations of FGFR inhibitors and BET inhibitors on RT112 and MGH-U3 cell viability

(A) MGH-U3 and RT112 cells were treated for 72 h with a range of doses of (+)-JQ1 and PD133074 separately or together. Cell viability was measured with CellTiter-Glo. **(B)** Mathematical analysis of cell viability data obtained with (+)-JQ1 and PD133074 drug combinations using SynerDrug (https://github.com/bioinfo-pf-curie/synerdrug) and the Loewe Additivity law. The combination index (CI) are shown. 0<CI<1 indicates a synergistic effect; CI=1 indicates an additive effect and CI>1 indicates an antagonistic effect.
