# Supplementary figures and images for: An FGFR3/MYC positive feedback loop provides new opportunities for targeted therapies in bladder cancers
Source: EMBO Mol Med. 2018 Feb 20;10(4):e8163. doi: 10.15252/emmm.201708163 (PMC5887543; doi:10.15252/emmm.201708163)

Source Data – Appendix Figure S3A

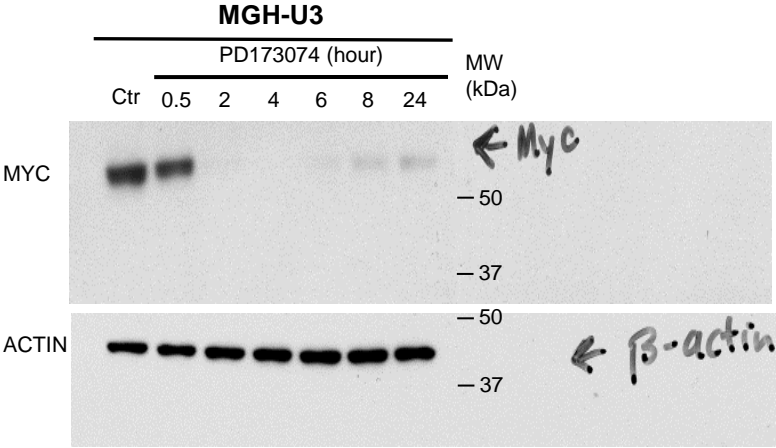

Source Data – Appendix Figure S3B

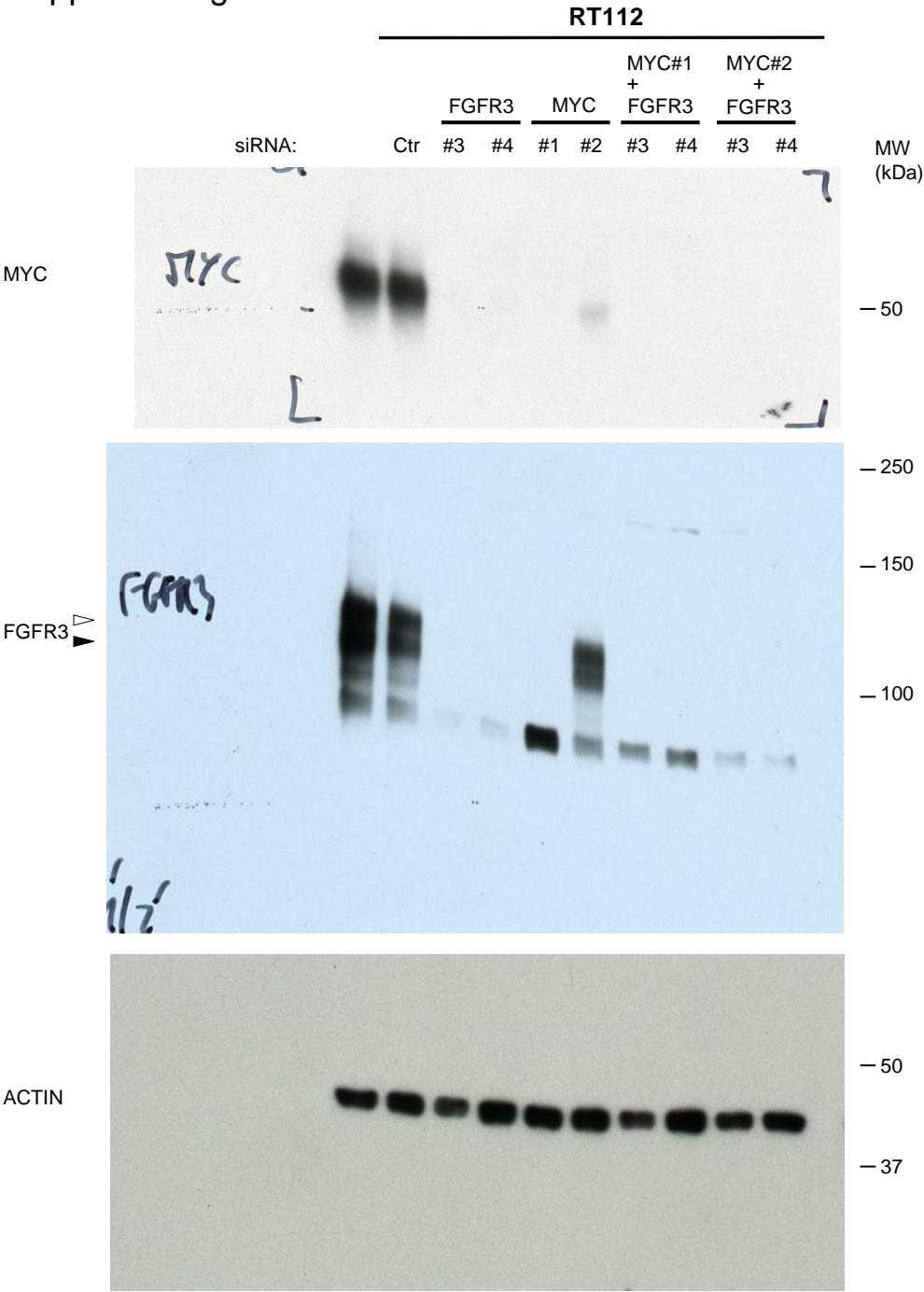

Supplement: Supplementary file 4 — Source Data for Appendix [file EMMM-10-e8163-s007.zip › Source_Data_Appendix_figures/Source_Data_Appendix_Figure_S3.pdf]

## Source Data – Appendix Figure S7

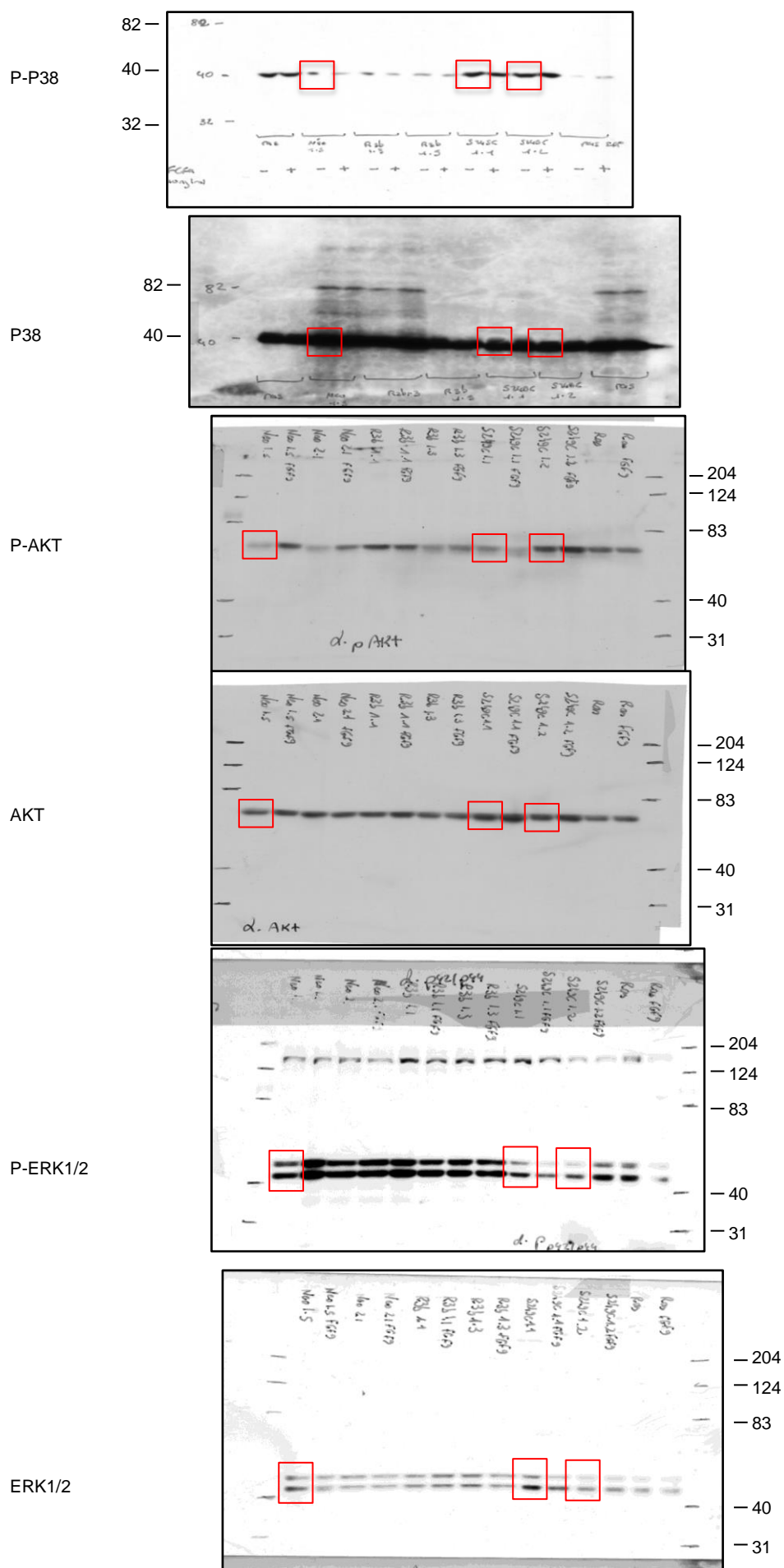

Supplement: Supplementary file 4 — Source Data for Appendix [file EMMM-10-e8163-s007.zip › Source_Data_Appendix_figures/Source_Data_Appendix_Figure_S7.pdf]

Source Data – Figure 1G

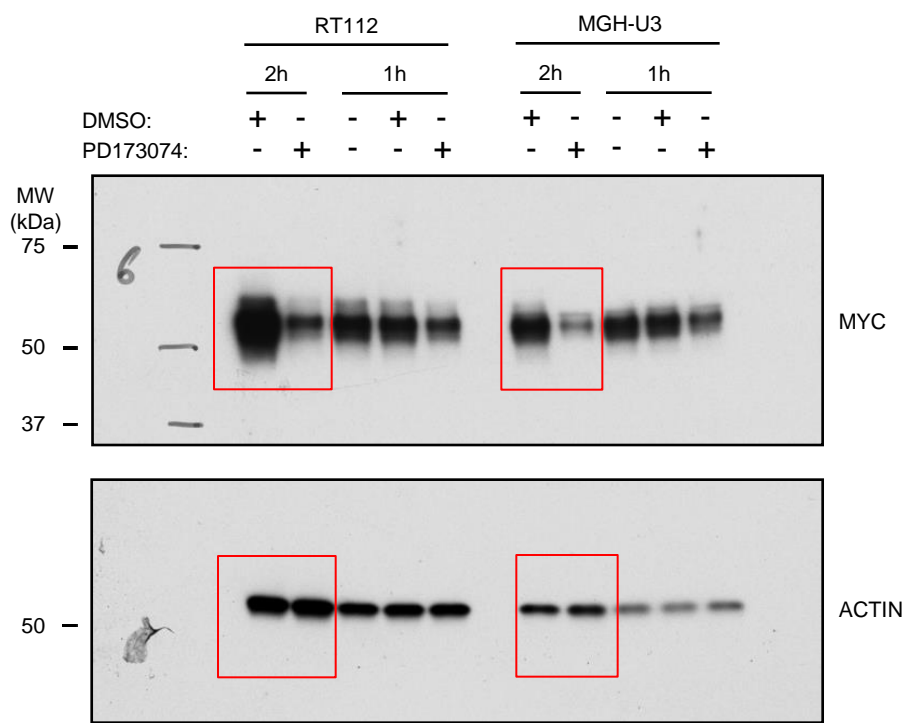

Supplement: Supplementary file 6 — Source Data for Figure 1 [file EMMM-10-e8163-s004.pdf]

Source Data – Figure 2E

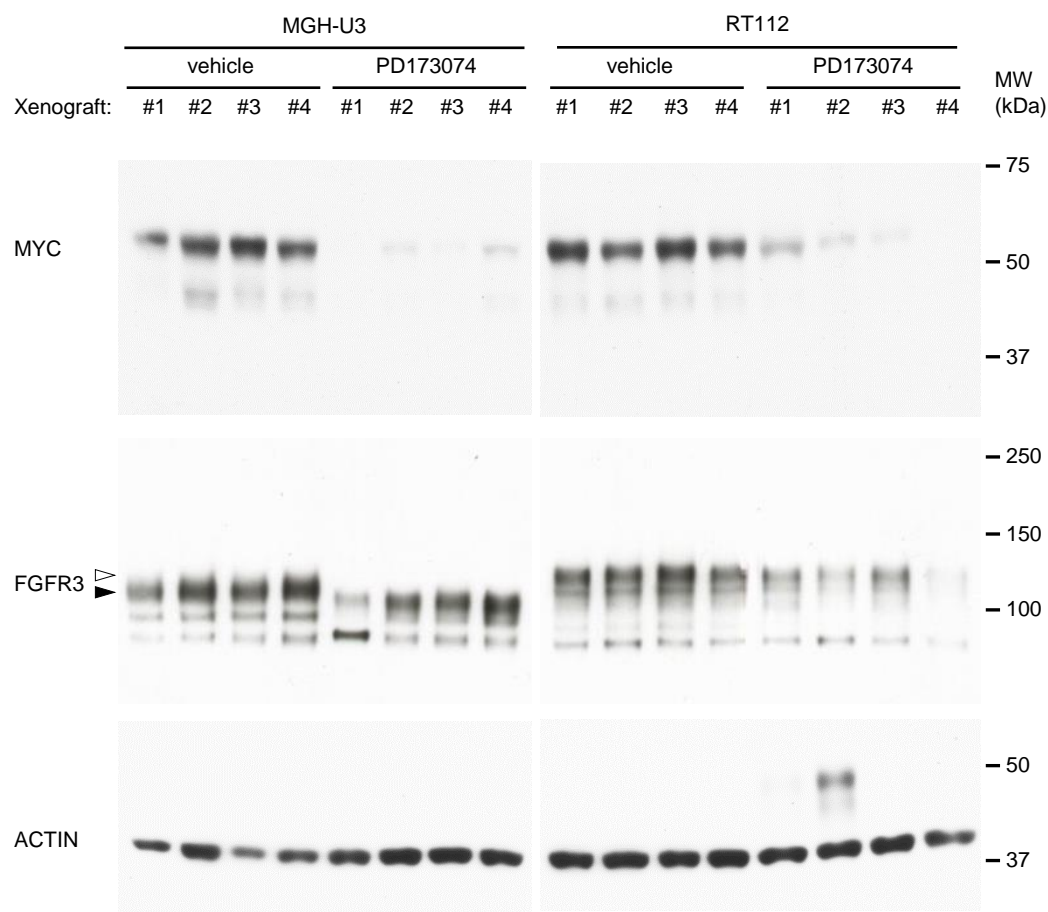

Source Data – Figure 2F

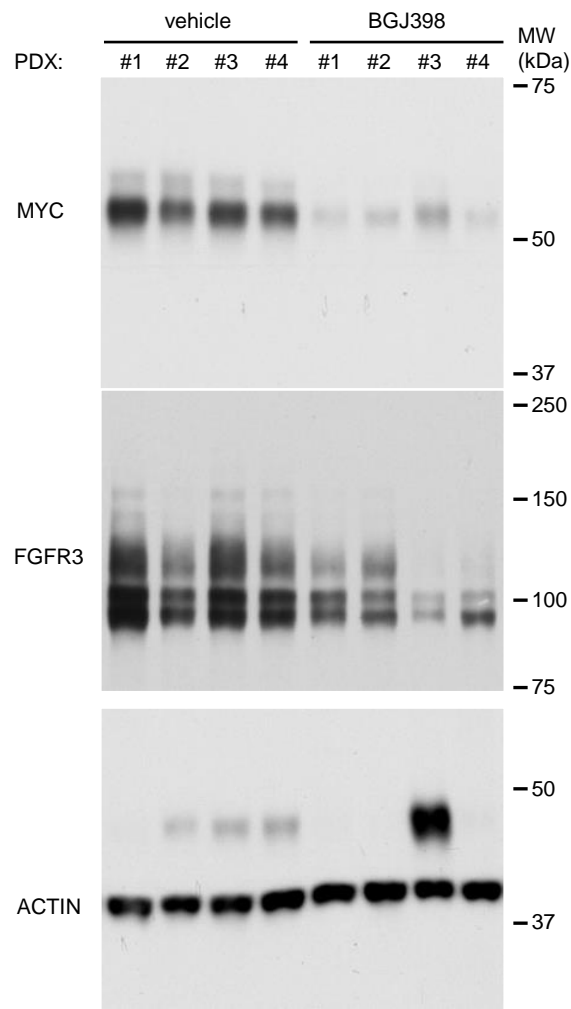

Supplement: Supplementary file 7 — Source Data for Figure 2 [file EMMM-10-e8163-s005.pdf]

Source Data – Figure 5B

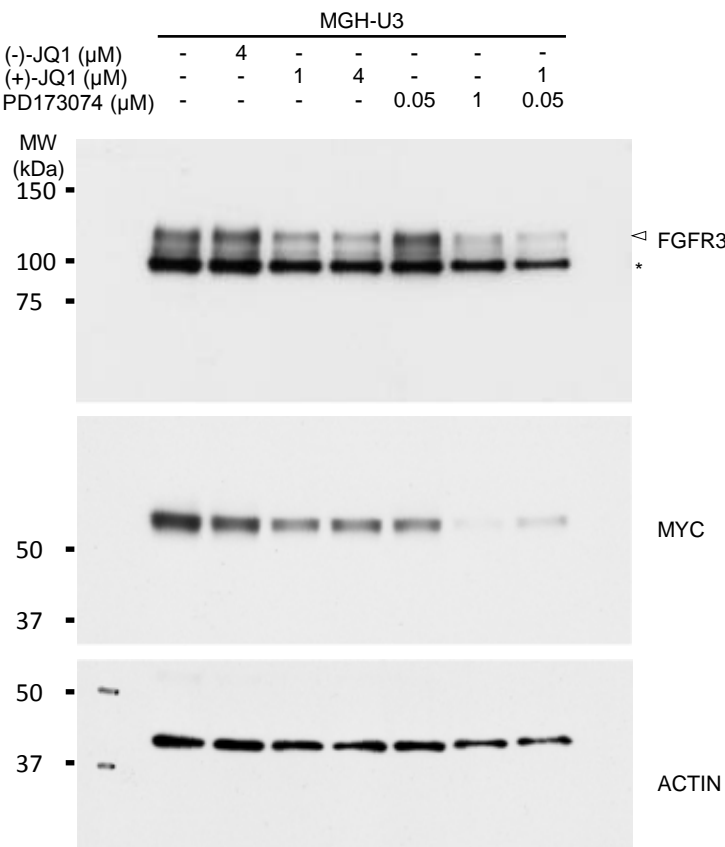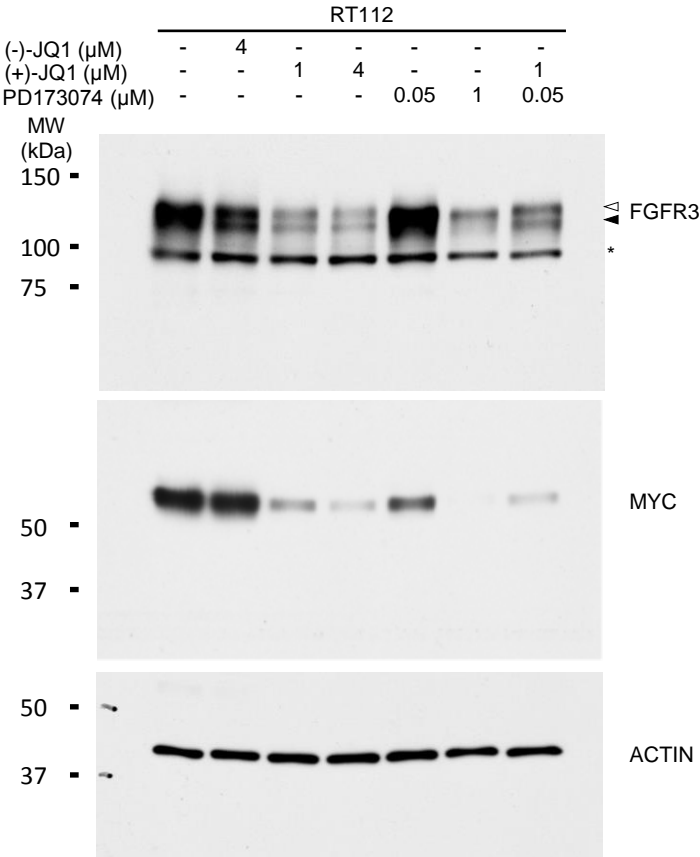

Supplement: Supplementary file 8 — Source Data for Figure 5 [file EMMM-10-e8163-s006.pdf]
